# Complete intracranial migration of ventriculoperitoneal shunt – a case report

**DOI:** 10.1097/RC9.0000000000000420

**Published:** 2026-04-01

**Authors:** Bizuayehu Asefa Tarekegn, Yemisirach Bizuneh Akililu, Tadelu Mekonnen Tsegaw, Yeabsira Fekadie Yihunie

**Affiliations:** Neurosurgery Division, Department of Surgery, Addis Ababa University, Addis Ababa, Ethiopia.

**Keywords:** case report, endoscopic retrieval, hydrocephalus, shunt complications, shunt migration, ventriculoperitoneal shunt

## Abstract

**Introduction:**

Hydrocephalus is commonly managed with ventriculoperitoneal shunt (VPS) placement. While effective, it carries risks such as malfunction, infection, and migration. Complete intracranial migration is a rare complication occurring in 0.1%–0.4% of cases.

**Case Presentation:**

A 5-month-old female infant presented with lethargy and vomiting three months after left Keen’s point VPS insertion. Imaging confirmed complete intracranial migration of the entire shunt apparatus into the ventricles. The shunt was retrieved endoscopically, followed by insertion of a new VPS contralaterally.

**Discussion:**

Proximal shunt migration is linked to factors such as wide burr holes, large dural openings, and insecure fixation. Endoscopic removal is standard, with new shunt placement if no infection is present. Preventive measures should be prioritized, and long-term monitoring is essential for detecting complications.

**Conclusion:**

Complete intracranial VPS migration is an uncommon complication that necessitates prompt intervention. Our experience underscores that adherence to strict surgical principles can substantially prevent its occurrence.

## Introduction

Hydrocephalus (HCP) is an increase in cerebrospinal fluid (CSF) volume due to impaired absorption, overproduction, or obstruction of normal CSF flow^[^1^]^. Ventriculoperitoneal shunt (VPS) insertion is the preferred management strategy^[^2,3^]^. Despite its widespread use, VPS is associated with multiple complications, which occur more frequently at the distal than the proximal end. Reported distal-end complications include shunt infection, obstruction, catheter migration, segmental breakage or disconnection, and CSF pseudocyst formation^[^4,5^]^.

Shunt migration is defined as the unintended movement of part or the whole of a shunt system from its original position into a new compartment. While distal migration into the thoracic or abdominopelvic viscera is more common, proximal migration into the cranial cavity is rarely reported. The overall incidence of shunt migration is approximately 0.1%–8.6% of cases. Total migration of the shunt into the cranial cavity is rare, accounting for 0.1%–0.4% of total migrations. It is reported more commonly in children than in adults^[^6–8^]^.

Patients with proximal shunt migration typically present with signs and symptoms of shunt malfunction, such as nausea, vomiting, headache, altered mental status, and seizures. Diagnosis is confirmed with imaging, including X-ray, CT, or MRI. Surgical management is the cornerstone of treatment for proximal shunt migration, involving removal of the migrated shunt system and insertion of a new VPS^[^9,10^]^.

HIGHLIGHTS

- Ventriculoperitoneal shunt (VPS) insertion is the preferred and most common option for the management of hydrocephalus.
- Proximal shunt migration is a rare complication of VPS insertion.
- The diagnosis of shunt migration is with typical symptoms of shunt malfunction coupled with radiologic confirmation with X-ray or CT scan.
- Management involves endoscopic removal of the migrated shunt apparatus with insertion of a new VPS.
- Long-term follow-up is crucial to monitor for recurrence and associated complications.

In this report, we describe a 5-month-old female infant with complete intracranial shunt migration.

This case report has been prepared in accordance with the SCARE guidelines^[^11^]^.

## Case presentation

A 5-month-old female infant was diagnosed with lumbar myelomeningocele immediately after birth, which was repaired at 48 hours of age. Following the repair, she developed progressive head enlargement due to hydrocephalus.

A left Keen’s point VPS was inserted at two months of age with an uneventful intraoperative and postoperative course. The dura was opened in linear fashion, and the shunt was fixed to the periosteum with 2-0 silk suture. However, at five months of age, she presented with vomiting of ingested material, feeding difficulty, and altered mental status of 2 days’ duration. On physical examination, she was lethargic, and the shunt system was not palpable at its insertion site at left Keen’s point.

**Figure 1.**
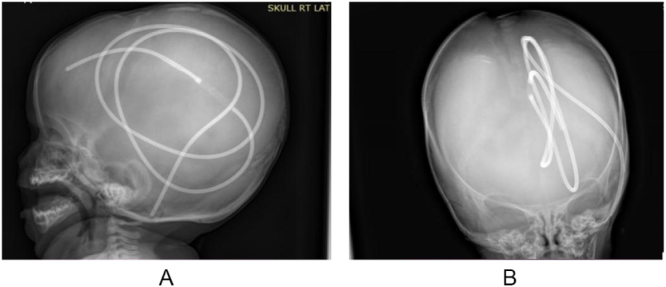
Lateral (A) and anteroposterior (B) skull X-ray images reveal migration of both proximal and distal segments of the ventriculoperitoneal shunt into the intracranial cavity with no disconnection between the two segments.

**Figure 2.**
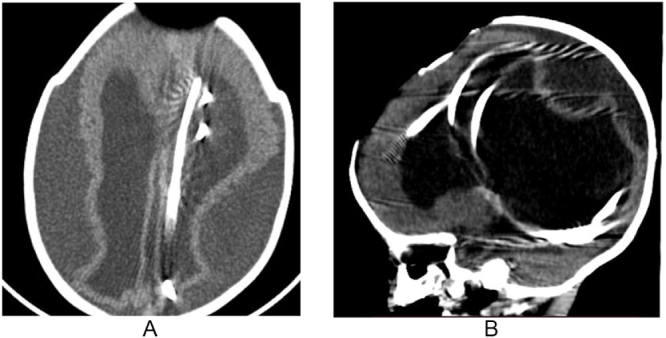
**A**xial CT (A) and sagittal (B) CT scan show migration of the shunt into the left lateral ventricle, dilation of the ventricular system, thin cortical mantle, and bilateral subdural collections.

Skull X-ray revealed complete intracranial migration of both the cranial and distal segments of the shunt apparatus (Fig. 1A, B). Brain CT scan confirmed this finding and showed associated ventricular enlargement and bilateral subdural collections (Fig. 2A, B). Preoperative CSF analysis revealed a WBC count of 2/mm^3^, protein 20 mg/dL, and glucose 40 mg/dL. Both gram stain and culture were negative.

Endoscopic retrieval was performed through the original burr hole. Intraoperatively, the original burr hole was found to be wide (1.5 cm), and the 2-0 silk anchoring suture had avulsed from the periosteum. Using endoscopic forceps, the hardware was retrieved without significant parenchymal injury.

Intraoperative manipulation resulted in lightly blood-stained CSF. To prevent blood products from obstructing the new shunt, an external ventricular drain (EVD) was placed for 3 days until the CSF cleared. A new Chhabra VPS was then inserted at right Keen’s point using a 1-cm burr hole and a 5-mm linear dural opening. The valve was securely fixed to both the periosteum and galea.

The patient was discharged on postoperative day 6. Follow-up evaluations at 1, 3, and 6 months showed complete resolution of symptoms.

## Discussion

VPS placement is one of the most common neurosurgical procedures, yet it is associated with numerous complications. Shunt migration is rare, and total intracranial migration is particularly uncommon, with only a few cases reported in the literature^[^7,9^]^.

The mechanisms of cranial migration are not fully understood, but factors thought to contribute to proximal shunt migration can be categorized into patient-related, shunt-system, and technical factors. For several reasons, pediatric patients are more vulnerable to shunt migration than adults^[12]^. A thin cortical mantle, enlarged ventricles, and wide fontanelles reduce intracranial pressure, while concomitantly elevated peritoneal pressure establishes a gradient that may facilitate proximal migration. This risk is further compounded by anatomical factors, including the short distance between the peritoneum and cranium and forceful, uncontrolled head movements. In addition, rapid growth in early life may lead to distal catheter detachment^[^13,14^]^.

Among technical factors, a large burr hole, wide dural opening, and improper shunt fixation are frequently implicated^[9]^. Rare reports also describe traumatic intracranial migration of a VPS^[^15^]^.

When an endoscope is available, removal of the migrated shunt system is typically performed through the existing burr hole or a new burr hole at Kocher’s point for simplicity. Some case reports describe leaving the shunt in situ, which was well tolerated; however, due to risks of fatal outcomes or infection, removal is generally recommended^[^7,12^]^.

In our case, the patient’s young age, combined with a wide burr hole and thin cortical mantle, likely contributed to the migration. Furthermore, as noted by Malhotra *et al*, the Chhabra shunt may be more prone to intracranial migration owing to its soft, cylindrical chamber design^[16]^. This underscores the importance of double anchorage to provide redundant security. The initial procedure used a single periosteal suture, which had detached. For the revision, we employed a more robust fixation technique by anchoring the valve to both the periosteum and the overlying galea to prevent recurrence.

The new shunt was placed on the contralateral side to provide a fresh surgical site. This allowed a more precise burr hole and limited dural incision, creating a more secure environment for the new hardware compared with the original dilated site. Preventive measures should focus on these technical aspects: using a small burr hole, minimizing dural opening, and ensuring firm double anchoring at the cranial site^[^16,17^]^. Long-term follow-up is essential to detect recurrent migration, repeated infections, or shunt malfunction.

## Conclusion

Complete intracranial VPS migration is a rare complication influenced by both physiological and surgical factors. The risk of migration can be minimized by using a small burr hole and dural incision. The shunt should be secured to both the periosteum and overlying galea.

## Data Availability

All data generated or analyzed during this study are included in this article.
